# Lymphocyte counts may predict a good response to mesenchymal stromal cells therapy in graft versus host disease patients

**DOI:** 10.1371/journal.pone.0217572

**Published:** 2019-06-12

**Authors:** Liad Hinden, Mordechai Avner, Polina Stepensky, Reuven Or, Osnat Almogi-Hazan

**Affiliations:** Department of Bone Marrow Transplantation, Hadassah-Hebrew University Medical Center, Jerusalem, Israel; European Institute of Oncology, ITALY

## Abstract

Steroid-resistant GvHD is one of the most significant causes of mortality following allogeneic Hematopoietic Stem Cell Transplantation (HSCT). Treatment with mesenchymal stromal cells (MSC) seems to be a promising solution, however the results from clinical studies are still equivocal. Better selection of candidate patients and improving monitoring of patients following MSC administration can increase treatment effectiveness. In order to determine which characteristics can be used to predict a good response and better monitoring of patients, blood samples were taken prior to therapy, one week and one month after therapy, from 26 allogeneic HSCT patients whom contracted GvHD and were treated with MSCs. Samples were examined for differential blood counts, bilirubin levels and cell surface markers. Serum cytokine levels were also measured. We found that the level of lymphocytes, in particular T and NK cells, may predict a good response to therapy. A better response was observed among patients who expressed low levels of IL-6 and IL-22, Th17 related cytokines, prior to therapy. Patients with high levels of bilirubin prior to therapy showed a poorer response. The results of this study may facilitate early prediction of success or failure of the treatment, and subsequently, will improve selection of patients for MSC therapy.

## Introduction

Allogeneic Hematopoietic Stem Cell Transplantation (HSCT) enable treatment of a number of hereditary and hematological diseases, however the most common implementation of HSCT is for the treatment of hematological malignancies. The anti-tumor effect of allogeneic transplants relies not only on chemical conditioning, but also on the action of the graft against the tumor, known as the graft versus leukemia effect (GvL).

Unfortunately, the immune effect of the graft is not limited to the tumor alone, and in many cases an immune response towards the patient’s body develops, known as graft versus host disease (GvHD). GvHD may appear as an acute or chronic disease. Traditionally, the acute disease was defined as appearance of the disease during the first 100 days after the transplant. However, the accepted practice today is to classify GvHD according to the organs involved and the characteristics of the disease. [1,2]

The pathogenesis of acute GvHD starts with tissue damage caused by conditioning and exposure of the immune system to bacterial antigens, which leads to a release of inflammatory cytokines and activation of the patient’s antigen presenting cells (APCs). Then, the APCs stimulate the T cells originating from the graft. Acute GvHD is mediated mainly by Th1 cells and by Th17 cells. Treg cells are able to control the inflammatory process and weaken it. Eventually, the activated immune cells cause damage to the patient’s tissues.[3] Chronic GvHD is initiated by naive T cells, differentiating predominantly within highly inflammatory T-helper 17/T-cytotoxic 17 and T-follicular helper paradigms with consequent thymic damage and impaired donor antigen presentation in the periphery. This leads to aberrant T- and B-cell activation. [4]

All patients undergoing an allogeneic bone marrow transplant also undergo preventative treatment via immunosuppressive therapy to prevent GvHD. Unfortunately, less than half those suffering from acute GvHD respond fully to treatment.[5] The prognosis of patients who develop GvHD after HCT and do not respond to primary therapy is poor. Numerous strategies to treat these patients with second-line therapies have been undertaken, aiming at inactivation of alloreactive donor T lymphocytes or NK cells, host antigen-presenting cells, cytokines, or cytokine receptors, or at tissue repair in the recipient. Overall, results have been disappointing. [6]

One of the treatments at the frontier of current research in this field is administration of mesenchymal stromal cells (MSCs). MSCs are multipotent cells that belong to the bone marrow stroma.[7] MSCs have low immunogenicity, and there is no need for tissue-matching between the donor and the recipient when they are administered as cell therapy.[8] In addition MSCs also have a mitigating influence on the activity of immune cells. For example, MSCs inhibit activation and proliferation of T lymphocytes in various mechanisms [9], among them they suppress secretion of the inflammatory cytokines and promote secretion of IL-10 that accompanies differentiation to Treg cells. [10]

Broad research was conducted in an attempt to understand MSC’s clinical potential. However, despite the positive initial results, a number of additional studies reported mixed results. [11,12,13]

The objective of our study is to identify the immune, hematological, and biochemical parameters that characterize the population of GvHD patients responding to MSC therapy, relative to non-responding patients.

Identifying the characteristics shared by patients who responded to treatment may facilitate early prediction of success or failure of the treatment, and subsequently improve selection of patients for treatment. In addition, identification of changes in the immune system in response to the treatment, that appear together with clinical improvements of the patients, may provide a basis for better follow-up and monitoring during the course of treatment, and may be important for understanding the biological mechanism for mesenchymal cells effect in GvHD.

## Materials and methods

### 1. Study population

The study included all the patients receiving allogeneic HSCT which contracted GvHD and were treated with MSCs in the Department for Bone Marrow transplantation at the Hadassah Medical Center, between November 2011 and February 2014. The Characteristics of the patients population are listed in **Table 1.** All the patients suffered from steroid-resistant GvHD. All the tests in this study were done as a part of patient monitoring. The collection of data from the patients’ files was performed under institutional approval in accordance with the declaration of Helsinki.

**Table 1:** Patients characteristics.

GvHD grade	sex	Age	Indication for BMT	Disease Status at BMT	Conditioning Protocol	Donor	Graft Source	GvHD type	Organ involvement	Days after BMT
1	Female	>18	AML	Active	MA	MUD	PB	Acute	S,G	**78**
1	Male	<18	SCN	Non-Malignant	MA	MUD	BM	Acute	S,G,L	**48**
2	Female	>18	AML	Remission	MA	MRD	PB	Chronic	S,O	**1074**
2	Female	<18	NHL	Remission	MA	MRD	BM	Chronic	S,G,O	**216**
2	Male	>18	ALL	Active	RIC	MMUD	PB	Acute	S,G,L,O	**41**
3	Male	>18	ALL	Active	MA	MMUD	PB	Acute	S,G,L	**70**
3	Female	<18	AML	Active	MA	MMUD	BM	Acute	S,G,L	**133**
3	Male	<18	Hurler	Non-Malignant	MA	MMUD	UCB	Acute	S,G	**27**
3	Male	>18	AML	Remission	MA	MRD	PB	Acute	S,G	**29**
3	Female	>18	AML	Remission	MA	MUD	PB	Chronic	S,G	**143**
3	Male	<18	CML	Active	MA	MMUD	PB	Acute	S,G	**28**
3	Male	<18	AML	Remission	MA	MUD	BM	Acute	S,G	34
3	Female	>18	AML/MDS	Remission	RIC	MRD	PB	Acute	S,G	106
4	Male	>18	NHL	Active	RIC	MMUD	PB	Acute	S,G	**74**
4	Male	>18	AML	Remission	RIC	MUD	PB	Acute	S,G	**86**
4	Female	>18	AML	Active	MA	MUD	PB	Acute	S,G	**31**
4	Female	>18	AML	Remission	MA	MMUD	UCB	Acute	G	**42**
4	Male	>18	AML	Remission	MA	MUD	PB	Chronic	S,G,L	**105**
4	Female	<18	LAD	Non-Malignant	MA	MMUD	BM	Acute	S,G,L	**51**
4	Male	>18	AML	Remission	RIC	MRD	PB	Acute	G	**94**
4	Male	>18	AML	Remission	MA	MMUD	PB	Acute	G	**43**
4	Male	>18	SAA	Non-Malignant	MA	MUD	BM	Acute	S,G	**70**
4	Male	>18	MDS	Active	MA	MMUD	PB	Acute	S,G	**36**
4	Female	>18	AML	Active	MA	MMUD	PB	Acute	S,G	**56**
4	Female	<18	Thalassemia	Non-Malignant	MA	MRD	BM	Acute	S,G	**46**
4	Male	>18	ALL	Remission	MA	MRD	PB	Acute	S,G	99

Abbreviations: GvHD—graft vs host disease; AML—acute myeloid leukemia; ALL–acute lymphoblastic leukemia; SCN—severe congenital neutropenia; NHL—non-Hodgkin Lymphoma; LAD—leukocytes adhesion deficiency; CML—chronic myeloid leukemia; SSA—severe aplastic anemia; MDS—myelodysplastic syndrome; MRD–matched related donor; MUD–matched unrelated donor; MMUD–mismatched unrelated donor; MA—myeloablation; RIC–reduced intensity conditioning. PB–peripheral blood; BM–bone marrow; UCB–umbilical cord blood. S- skin, G-gut, L- liver, O- other.

### 2. Study design

Blood samples taken on the day of MSC therapy, one day and one month after therapy were used for monitoring the levels of hemoglobin, number and composition of leucocytes and the number of platelets, as well as bilirubin levels. Additionally, flow cytometry and cytokines analysis were performed.

An evaluation of the response to therapy was conducted within a range of 4–31 months after MSC therapy by the doctor treating the patients. The patients were defined as “responding” or “not responding” to therapy according to the criteria for determining the severity of GvHD as determined by EBMT.[5]

### 3. Preparation and treatment with MSCs

Bone marrow cells were extracted and cultured as previously described [14], cells were frozen in a medium comprising of low-glucose DMEM with 80% FBS and 10% DMSO. The samples were partially defrosted, re-suspended in low-glucose DMEM, 35% FBS, 1% glutamine with added 1% of penicillin, streptomycin and nystatin (Biological Industries, Beit HaEmek, Israel). The cells were centrifuged, washed twice using normal saline and suspended in 15–20 ml normal saline prior to injection.

The cells were administered intravenously at a slow rate into a central or peripheral vein at a cell dose of 0.59 to 1.8 million cells per kg (median = 1.06).

### 4. Blood samples

Routine blood tests were taken as part of the accepted follow-up procedure. The blood samples were tested at the central laboratory of Hadassah Medical Center: Differential Complete blood counts (CBC) were performed using LH 750 Analyzer (Beckman Coulter, USA). Bilirubin level was measured (Cobas C Rosch/Hitachi, Germany). In addition, separate blood samples were taken for flow cytometry and for cytokine analysis.

### 5. Flow cytometry

Samples were centrifuged; the cells were re-suspended in a medium comprising DMSO 10% + FBS 20% + 70% cRPMI and frozen in liquid nitrogen. Subsequently, the cells were defrosted and suspended in FACS medium. Samples were stained with antibodies against: CD3, CD19 and CD56 (Beckman Coulter, Marseille, France) and analyzed with a Miltenyi MACSQuant FACS (Biotech, Germany). Data processing was conducted using FCS Express V3 software.

### 6. Cytokin analysis in serum

Serum was collected, and frozen at -80°C. Subsequently, the samples were defrosted, and the level of cytokines in the serum was measured using a human FlowCytomix Th1/Th2/Th9/Th17/Th22 13plex kit (eBioscience, San Diego, USA) according to the manufacturer’s instructions. The samples were analyzed with a aMACSQuant FACS (Miltenyi Biotech, Germany), and the levels of cytokines in the samples were calculated using FlowCytomix Pro software.

### 7. Statistical methods

In order to compare the relationship between qualitative variables and the research groups, we used the chi-square test or Fisher’s exact test. Comparison of a quantitative variable between two independent groups was done using the non-parametric Mann-Whitney U test. Comparison of the quantitative variable among three groups was done using the non-parametric Kruskal-Wallis test with multiple paired comparisons and correction of the significance levels according to the Bonferroni test. The Wilcoxon test was applied to test the change between two time periods for a quantitative variable. Non-parametric tests were used because of the small sample size. All the statistical tests were two-tailed, and a p-value of 0.05 or less was considered to be statistically significant.

### 8. Ethical considerations

MSC therapy for treatment of GvHD was authorized by the institutional Helsinki Committee. Treatment was authorized privately for each patient (IRB). All the tests in this study were done as a part of patient monitoring. The collection of data from the patients’ files was performed under institutional Helsinki approval.

## Results

### The effect of patients’ clinical characteristics on the type of response to therapy

Among the 26 patients who were examined, 13 patients were defined as responders and 13 patients were defined as non-responders. The survival rate of responder patients 40 days after therapy was significantly higher than among non-responder patients (**Table 2**).

**Table 2:** Characteristics of responder patients versus non-responder patients.

		Responding	Not responding	P value
Sex	Male	4 (69.2%)	6 (46.2%)	0.43
	Female	9 (30.8%)	7 (53.8%)	
Age	<18	6 (46.2%)	2 (15.4%)	0.2
	>18	7 (53.8%)	11 (84.6%)	
Indication for BMT	Malignant disease	11 (84.6%)	10 (76.9%)	>0.99
	Non-malignant disease	2 (15.4%)	3 (23.1%)	
Conditioning protocol	Full intensity	11 (84.6%)	10 (76.9%)	>0.99
	Reduced intensity	2 (15.4%)	3 (23.1%)	
Donor	MRD	4 (30.8%)	3 (23.1%)	0.78
	MUD	3 (23.1%)	5 (38.5%)	
	MMUD	6 (46.2%)	5 (38.5%)	
Graft Source	Peripheral blood	8 (61.5%)	9 (69.2%)	0.22
	Bone marrow	5 (38.5%)	2 (15.4%)	
	Umbilical cord blood	0 (0%)	2 (15.4%)	
Type of GvHD	Acute	11 (84.6%)	11 (84.6%)	>0.99
	Chronic	2 (15.4%)	2 (15.4%)	
GvHD grade	1 to 2	3 (23.1%)	2 (15.4%)	>0.99
	3 to 4	10 (76.9%)	11 (84.6%)	
Days after BMT (days)				0.39
Median (range)		46 (28–216)	74 (27–1074)	
Involvement of liver	With	4 (30.8%)	2 (15.4%)	0.64
	Without	9 (69.2%)	11 (84.6%)	
Survival 40 days after therapy	Yes	11 (84.6%)	4 (30.8%)	0.015

In parentheses–the proportion of patients among those responding or not responding to therapy. Abbreviations: MRD–matched related donor; MUD–matched unrelated donor; MMUD–mismatched unrelated donor.

A number of patient variables were examined to determine whether there is a relationship between said variables and the type of response to therapy (**Table 2**). No significant difference among groups was found with respect to the origin of the graft, the patient’s gender, indications for transplantation, conditioning protocol, donor, type and severity of GvHD, time from transplant until MSC therapy and the proportion of patients with involvement of the liver.

We note that although no significant difference in response to therapy was found between children and adults, the response rate among children was better than among adult patients, a trend that is in line with previous studies in this field.[15]

### Patients with high lymphocyte counts before MSC therapy responded better to therapy

One of the challenges in MSC therapy is the selection of suitable patients for therapy. In order to determine which characteristics can be used to predict a good response to therapy, blood samples were taken from all of the patients prior to therapy, and were examined for blood count, composition of white blood cells (differential count) and bilirubin levels. In addition cell surface markers were examined by FACS and serum cytokine levels were measured. The various examined characteristics were compared retrospectively between responder patients and non-responder patients.

**Fig 1:**
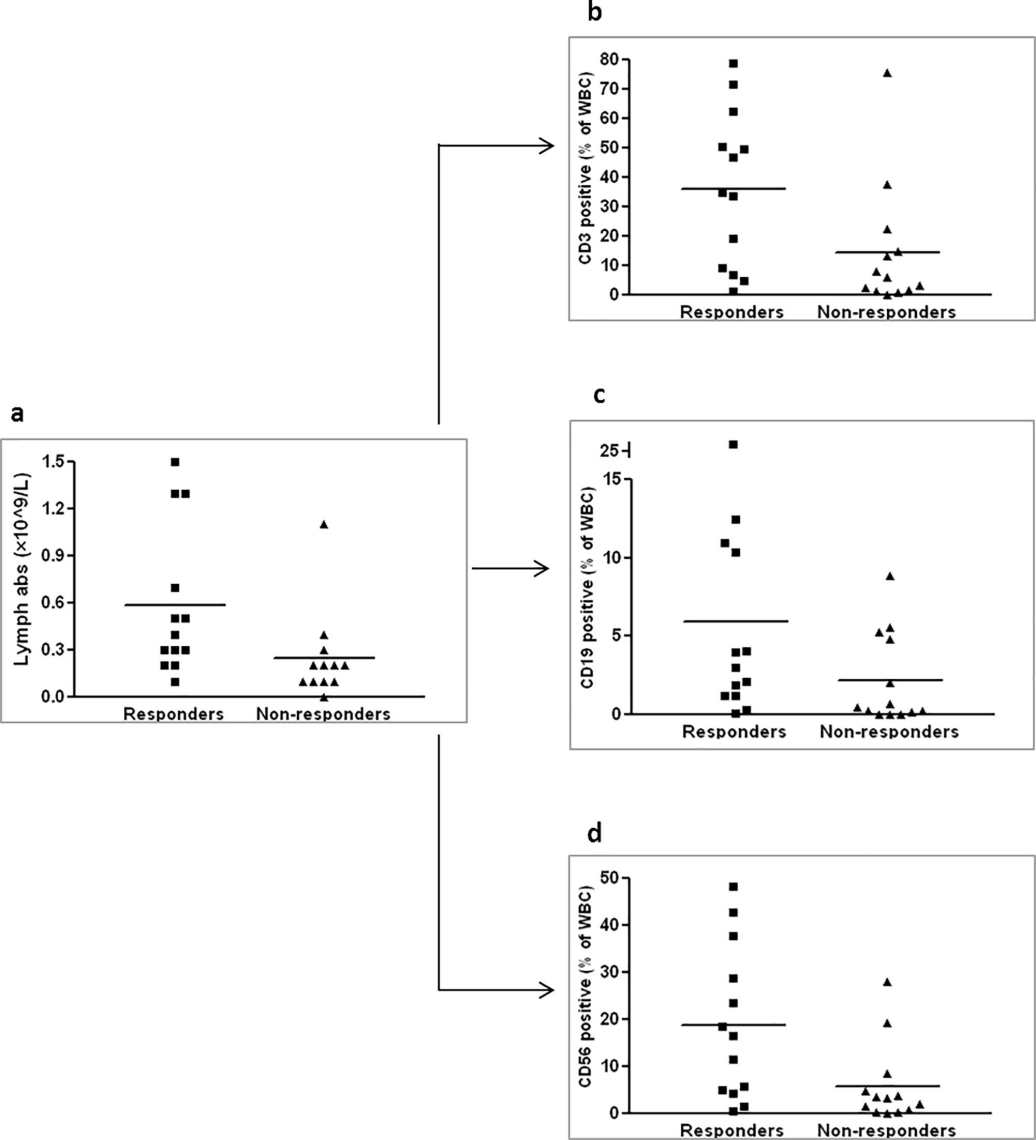
A high level of lymphocytes in peripheral blood prior to MSC administration among GvHD patients who responded to therapy. Blood samples were collected prior to therapy and blood count with differentials were examined. The lymphocyte level was higher in the responders. The difference between the groups is significant (P = 0.01) (a). Proportion of CD3 cells (P = 0.022) (b), proportion of CD19 cells (P = 0.64) (c) and proportion of CD56 cells (P = 0.01) (d) from total white blood cell (WBC) population as measured by flow cytometry.

We found that the lymphocyte count in the peripheral blood prior to therapy among responder patients is significantly higher than that of non-responder patients (**Fig 1A**). An examination of the cell populations by FACS demonstrated a higher proportion of T cells (CD3, **Fig 1B**) and NK cells (CD56, **Fig 1D**) among the total white blood cells of the responders. In addition, a higher proportion of B cells (CD19, **Fig 1C**) was also found, however this result was only marginally significant (P = 0.064).

### Increased lymphocyte level one month after MSC therapy in responder patients

**Fig 2:**
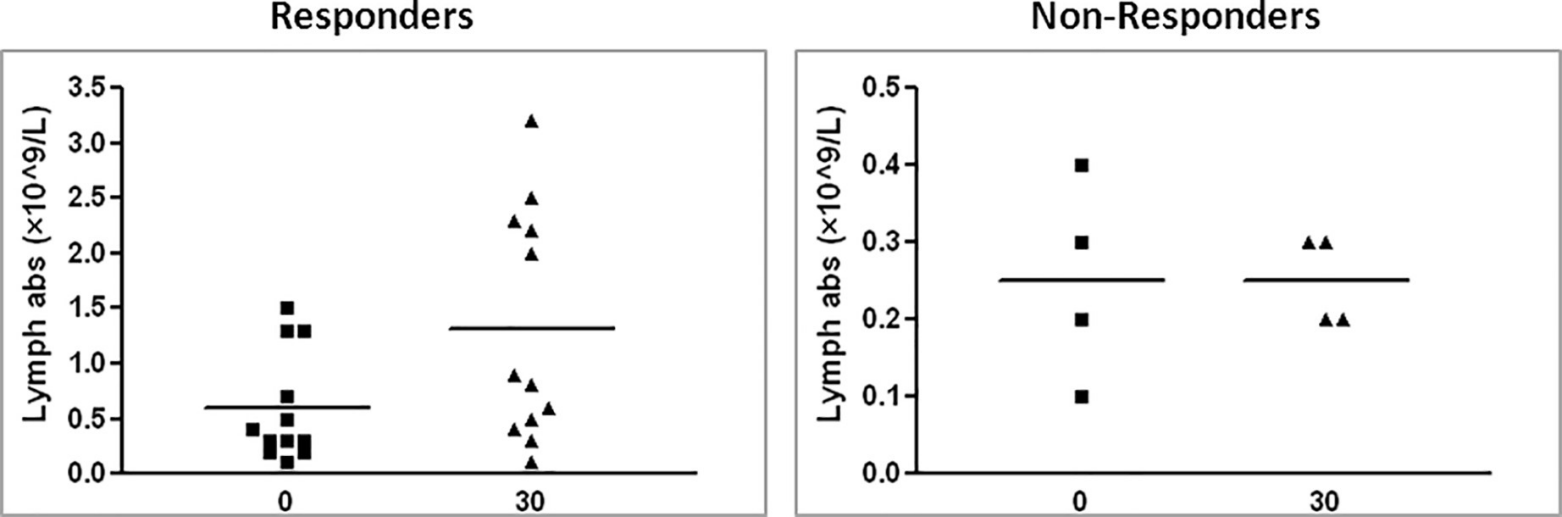
The change in lymphocyte levels 30 days after MSC therapy. Samples were taken from GvHD patients prior to MSC therapy and 30 days after therapy; blood count and differential count were examined. The change in responder patients appears on the left (p = 0.037) and among non-responder patients on the right (p = 0.74).

We further examined the change in the same characteristics in response to therapy. Blood counts from the day of therapy were compared to blood counts conducted one week and one month after therapy. No difference was observed after a week, however when comparing lymphocyte levels prior to therapy with those obtained one month after therapy in surviving patients, a significant increase could be observed among responder patients (**Fig 2**). This increase reinforces the higher initial level of lymphocytes in this group. A similar increase among non-responders was not observed; however, we note that the sample size of non-responders one month after therapy was relatively small (four patients) due to early mortality of these patients.

### Patients with a high platelet count prior to MSC therapy responded better to the therapy

**Fig 3:**
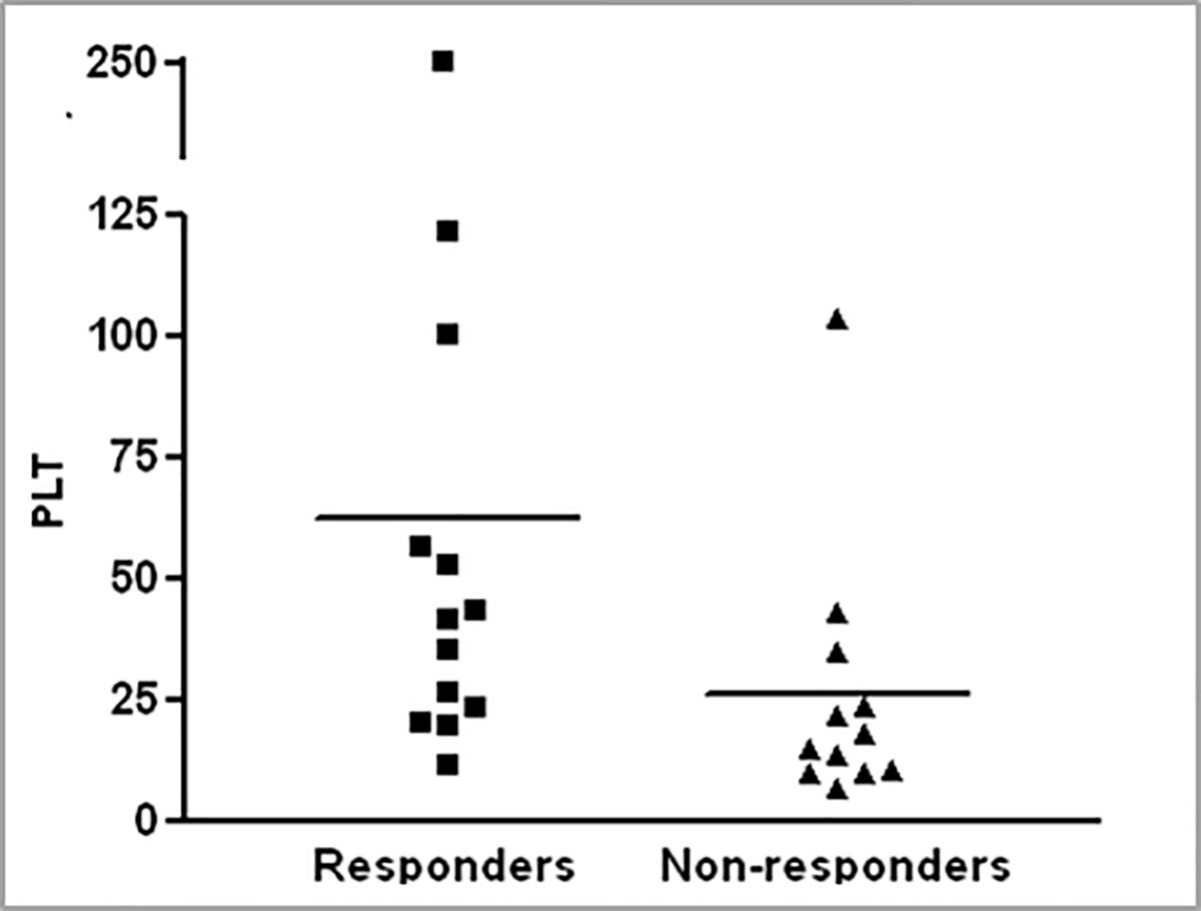
A high level of platelets in peripheral blood prior to MSC administration among GvHD patients who responded to therapy. Platelet levels prior to therapy in patients who responded to MSC therapy versus patients who did not respond (p = 0.01).

The level of platelets after allogeneic bone marrow transplantation is a very sensitive measure of the activity of the hematopoietic system. In many transplanted patients, platelet recovery may be delayed by several months. In the measurement of platelet levels prior to therapy, significantly higher levels were observed among patients who responded to MSC therapy (**Fig 3**).

### Anti-inflammatory cytokine profile in serum of responder patients

**Fig 4:**
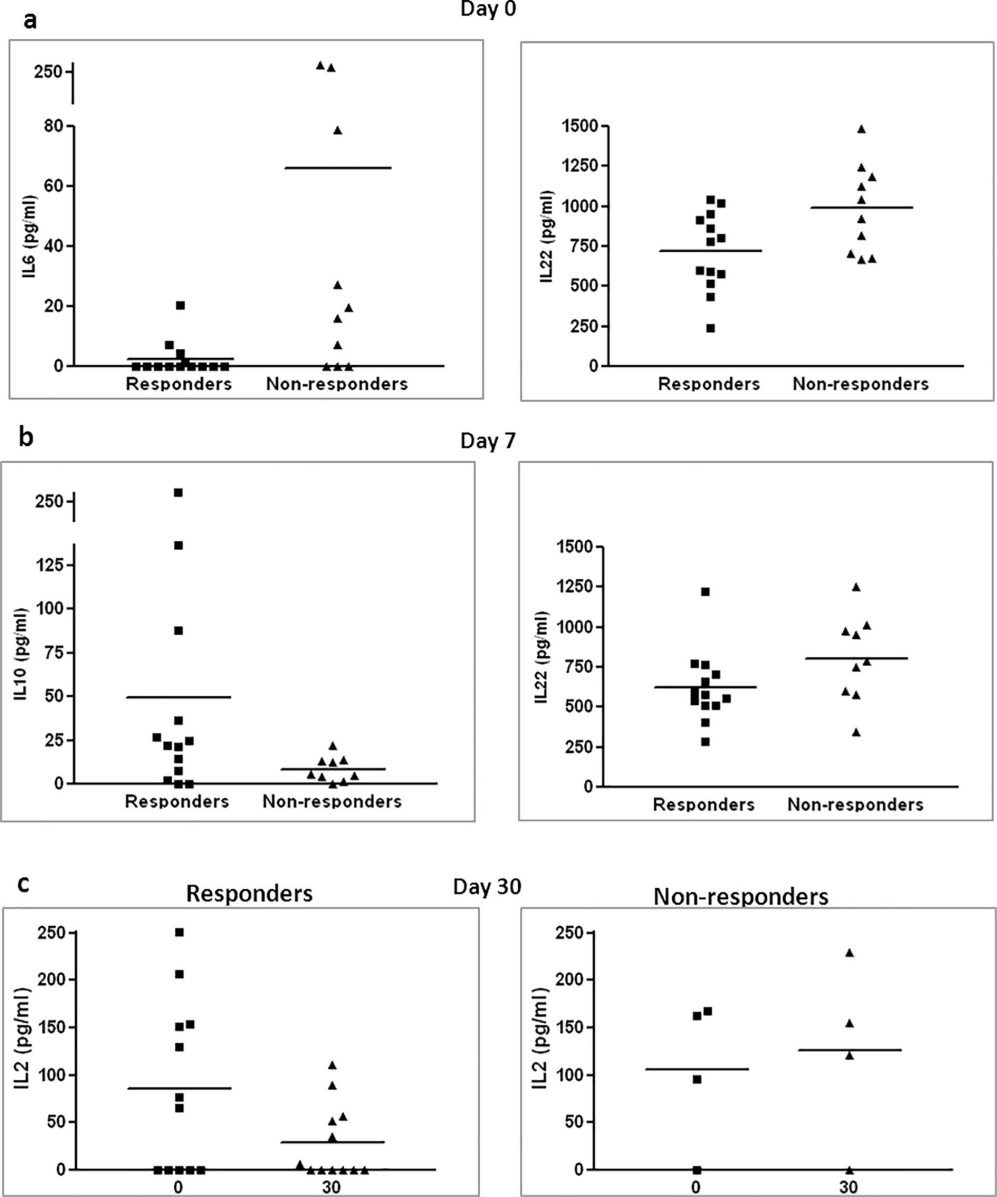
Cytokine profile of treated patients. Blood samples were collected prior to therapy, one week and one month after therapy. Samples were examined by cytokine array. Difference in levels of IL-6 (p = 0.03) and IL-22 (p = 0.026) among patient groups prior to therapy (a), difference in levels of IL-10 (p = 0.071) and IL-22 (p = 0.071) among patient groups one week after therapy (b). The change in IL-2 levels one month after cell therapy in responder patients (p = 0.04) and non-responder patients (p = 0.285) (c).

The level of cytokines in serum was also measured before and after therapy. Prior to therapy, we observed a lower level of IL-6, a known pro-inflammatory cytokine, and IL-22, which is secreted mainly by Th17 cells, among responder patients (**Fig 4A**). One week after therapy, we measured higher levels of IL-10, a known anti-inflammatory cytokine that is secreted by Treg and other cells, and lower levels of IL-22, in the serum of responders compared to non-responders (**Fig 4B**). When comparing levels of IL-2, an important component of the inflammatory response, prior to therapy and one month after therapy, a significant decrease was observed among responder patients but not among non-responder patients (**Fig 4C**).

#### High bilirubin levels prior to therapy in non-responder patients

**Fig 5:**
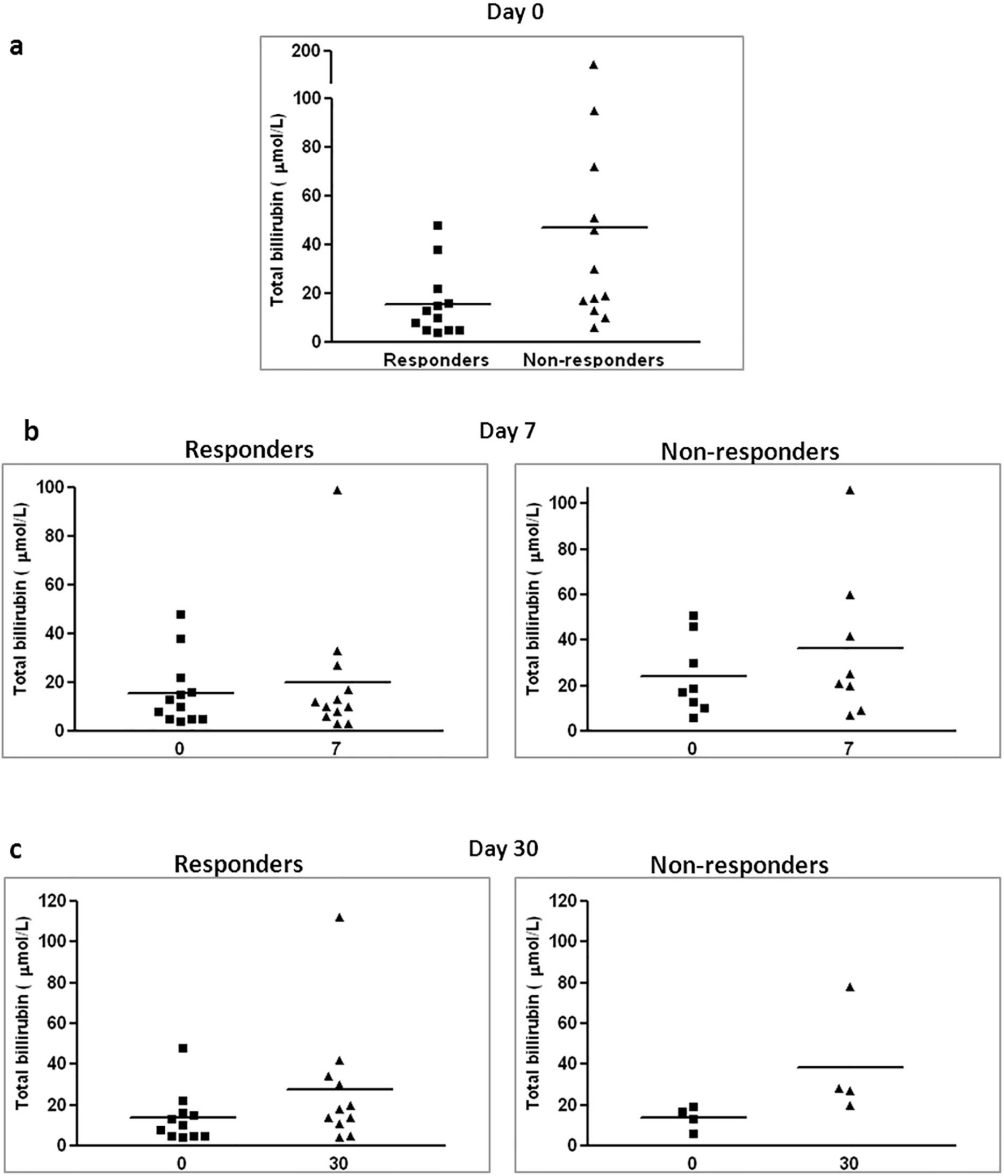
Bilirubin levels in treated patients. Blood samples were collected prior to therapy, one week and one month after therapy and blood bilirubin level was measured. Bilirubin levels prior to therapy in responder patients versus non-responder patients (a). Change in bilirubin level one week after therapy in non-responder patients on the right (p = 0.03) and in responder patients on the left (p = 0.798) (b). Change in bilirubin level one month after cell therapy in non-responder patients on the right (p = 0.066) and responder patients (p = 0.021) (c).

High levels of bilirubin in the blood of transplanted patients point to GvHD activity in the liver. When measuring bilirubin levels prior to therapy, a higher level of bilirubin was found among non-responder patients (**Fig 5A**). Subsequently, an increase in bilirubin levels was observed among non-responder patients, but not in responder patients, approximately one week after therapy in comparison to the levels measured prior to therapy (**Fig 5B**). A significant increase in bilirubin levels among responders was observed only one month after therapy. Among non-responders an increase in bilirubin was observed in the measurements conducted one month after therapy, however this was only marginally significant (p = 0.066), probably due to the small number of patients in this group that survived for a month after therapy (**Fig 5C**).

## Discussion

MSC therapy is an innovative and promising approach to treating GvHD following allogeneic HSCT, however recent studies in this field have presented mixed results. Therefore, it is very important to improve the effectiveness of therapy, identify suitable patient populations and strengthen our understanding of MSC’s mechanism of action.

This study examined immune, hematological and biochemical characteristics of GvHD patients who received MSC therapy. The different characteristics were compared between the populations of responder and non-responder patients, with the aim of finding characteristics that would enable better selection of patients for therapy as well as those that could provide a basis for better follow-up and monitoring of patients during therapy.

We found that the level of lymphocytes, in particular T and NK cells, may predict a good response to cell therapy. The increase in lymphocyte counts among responders was maintained even one month after therapy. Higher lymphocyte levels may indicate better recovery and acceptance of the graft. We found a similar relationship for rehabilitation of the hematopoietic system in general, as expressed by the platelet levels among transplanted patients.

MSC require an inflammatory environment for their immunomodulatory action [16]. In light of this, it is possible that the increase in therapy effectiveness among patients with high lymphocyte counts may arise from better activation of the MSC cells in these patients. A recent study conducted in our laboratory [17] demonstrated that pre-treatment of MSC cells using inflammatory cytokines may eliminate the requirement for an inflammatory environment in the patient.

Th17 cells contribute to the development of GvHD in both animal models and humans.[18] In this study a better response to MSC therapy was observed among patients who expressed low levels of IL-6 and IL-22 prior to therapy. IL-6 is an important cytokine for the development of the Th17 immune response, when IL-22 is expressed by these cells.[19] We may conclude that MSCs are more effective at treating GvHD, when the disease's characteristics are low levels of the Th17 immune response. Accordingly, one week after therapy a lower level of IL-22 was still observed among responder patients, a fact that may indicate a lower level of Th17 cell activity, also as a response to the therapy.

A number of studies have shown a relationship between MSC activity and the anti-inflammatory cytokine IL-10.[10,20] In this study, higher levels of IL-10 were observed one week after therapy among responder patients. IL-10 is secreted by a number of regulatory cells in the immune system. [21] Another cytokine affected by MSC therapy is IL-2. Following therapy there was a decrease in IL-2 levels among responder patients. IL-2 at high levels acts as an inflammation-promoting cytokine; however, at low levels it is essential for the development of regulatory T cells.[22] Together, these results strengthen the evidence for the contribution of Treg cells to the action of MSCs on GvHD.

A number of studies published in recent years examined the response of the immune system to MSC therapy. A study by Dander et al.[23] demonstrated a decrease in Th1 and Th17 cells and an increase in Treg cells in responder patients. These results are in line with the results of the present study. Another study [24] examined immune characteristics of GvHD patients who received cell therapy in comparison with patients who received a placebo, which found a shift of the immune response in the direction of Th2 and a decrease in the proportion of Th17 cells in treated patients. In a study that examined immune characteristics in patients with chronic GvHD who received MSC therapy [25], an increase in IL-10-secreting regulatory B cells was demonstrated. These results are also in line with the results presented here.

Te Boome et al. [26] examined biological indications that may predict a good response to MSC therapy in GvHD. In their study, the relationship between T and B cell levels prior to MSC therapy and the response to therapy was not significant; however, in their study, the sample size was small, and it is possible that the difference in results arose from the different sample sizes in the two studies; a larger study could strengthen the conclusions of both these studies.

In addition to parameters related to the immune system, we found that patients with high levels of bilirubin prior to therapy showed a poorer response. Even after therapy, these patients were characterized by a more rapid increase in bilirubin levels. High levels of bilirubin in patients after bone marrow transplantation may arise from GvHD in the liver, and it may be that the increase in bilirubin is an indicator for a bad prognostic response to therapy and an indication for development of liver disease even at the sub-clinical level. However, we must remember that an increase in bilirubin in patients following bone marrow transplantation has a broad differential diagnosis. In order to accurately identify the relationship between liver disease and the response to MSC therapy, there is a need for more comprehensive research.

The advantages of the research presented here, arises from the combination of clinical evaluation of laboratory variables, and the use of a range of methods for evaluating the immune system, as well as the examination of a large number of different types of variables. In addition, this study provides a broad picture of the response to therapy, by comparing patient characteristics before and during therapy.

The conclusions of the present study may provide a basis for the use of lymphocyte counts as a possible tool for selecting patients to receive MSC therapy for GvHD. With respect to the higher platelet levels among responder patients, this change may be related to recovery of the hematopoietic system from the transplant. Therefore, we may consider evaluation of recovery and graft acceptance as a condition for cell therapy. Similarly, this study demonstrates the importance of monitoring the Th17 immune response in patients before and during therapy. It may be possible in the future to characterize different populations of GvHD patients by the mixed immune response and to determine treatment accordingly.

This study also demonstrated, for the first time, the relationship between bilirubin levels and the response to MSC therapy, a finding that may be used as a tool for monitoring patients response to therapy.

The results of this study may facilitate early identification of responder patients and non-responder patients and development of research tools for evaluating cell activity and improving their action.

## Supporting information

S1 File (DOCX)
