# Supplementary figures and images for: Lymphocyte counts may predict a good response to mesenchymal stromal cells therapy in graft versus host disease patients
Source: PLoS One. 2019 Jun 12;14(6):e0217572. doi: 10.1371/journal.pone.0217572 (PMC6561566; doi:10.1371/journal.pone.0217572)

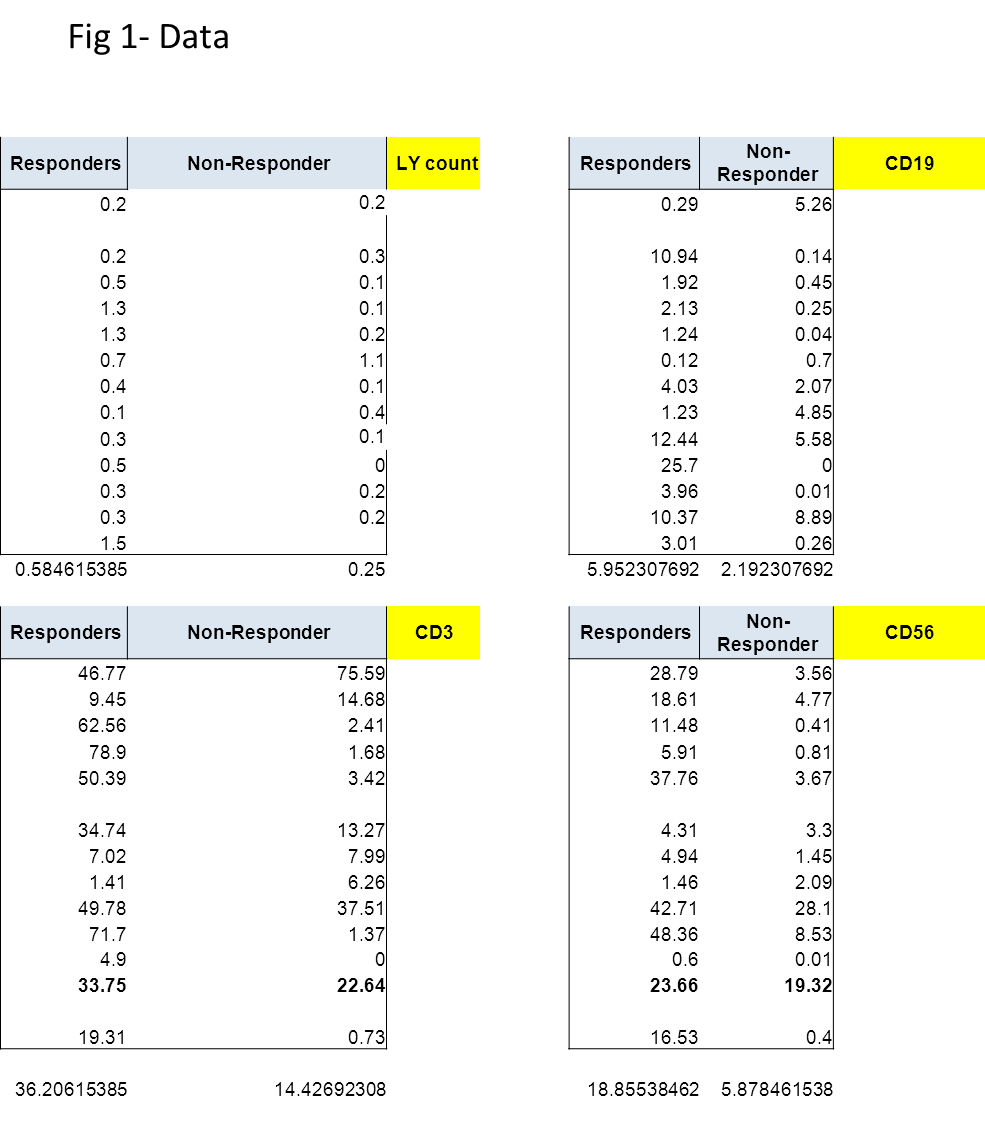


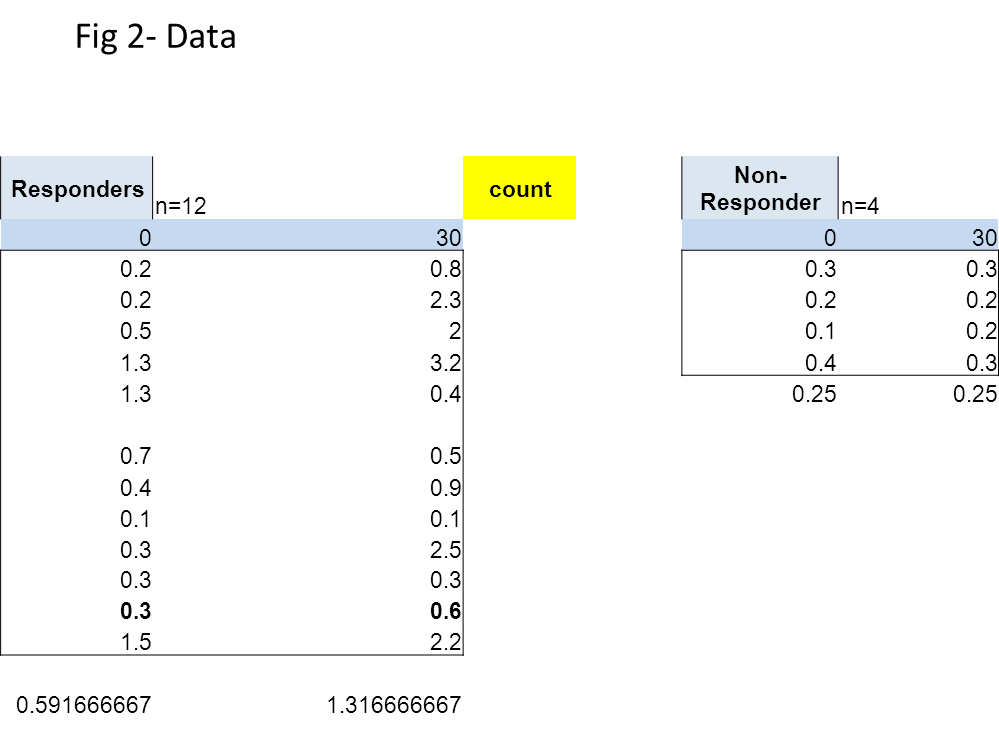


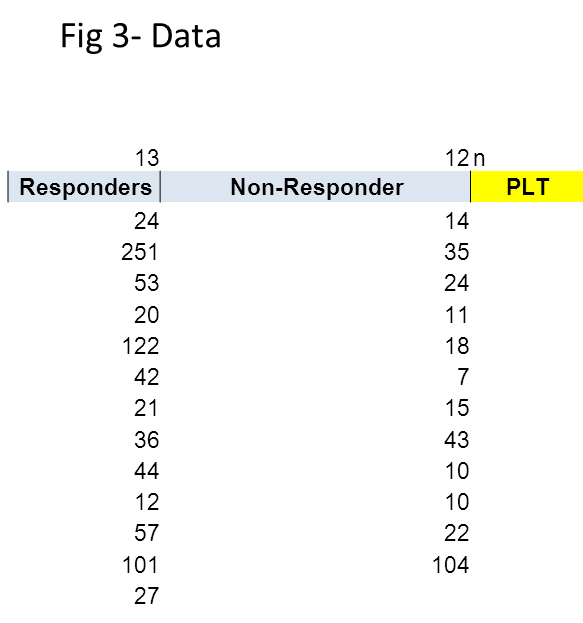


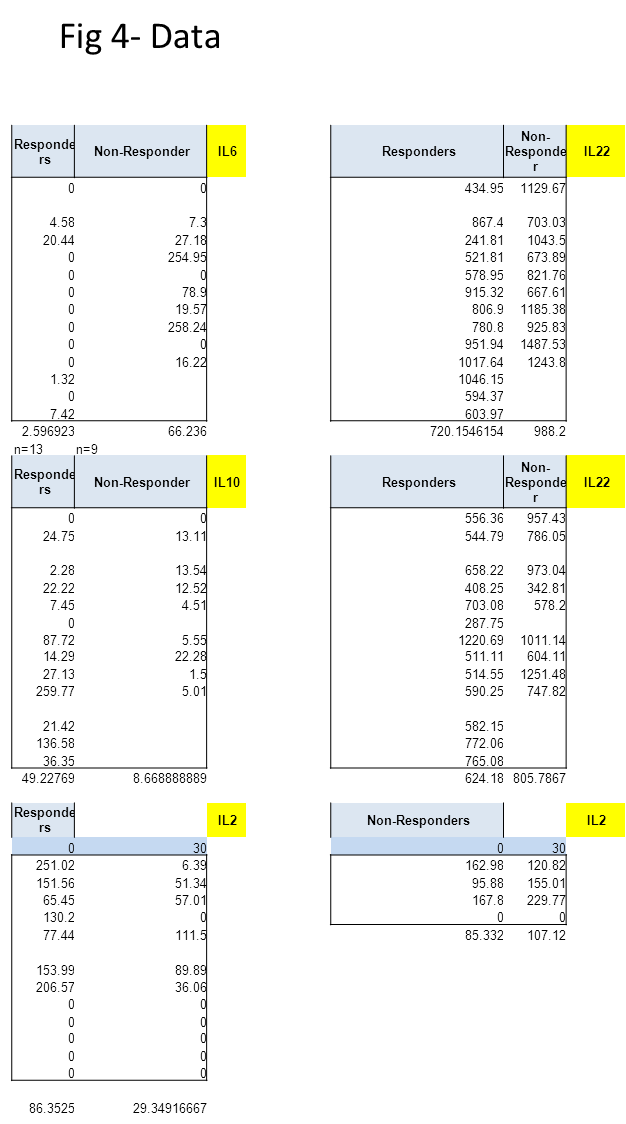


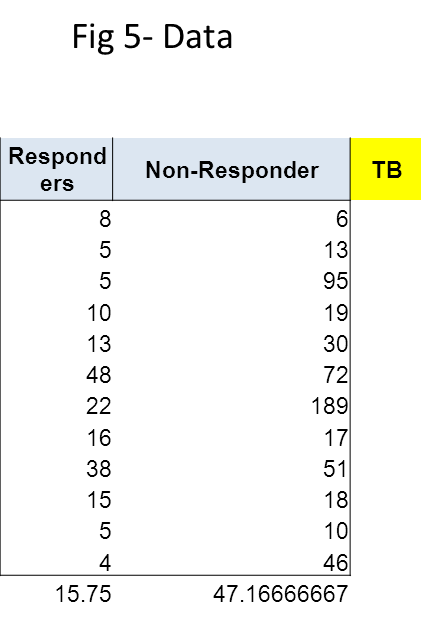

Supplement: S1 File — (DOCX) [file pone.0217572.s001.docx]
